# Concrete versus abstract forms of social concept: an fMRI comparison of knowledge about people versus social terms

**DOI:** 10.1098/rstb.2017.0136

**Published:** 2018-06-18

**Authors:** Grace E. Rice, Paul Hoffman, Richard J. Binney, Matthew A. Lambon Ralph

**Affiliations:** 1 Neuroscience and Aphasia Research Unit (NARU), University of Manchester, Manchester, UK; 2 Centre for Cognitive Ageing and Cognitive Epidemiology (CCACE), Department of Psychology, University of Edinburgh, Edinburgh, UK; 3 School of Psychology, University of Bangor, Wales, UK

**Keywords:** semantic cognition, conceptual knowledge, social cognition, face recognition, anterior temporal lobe

## Abstract

The anterior temporal lobes (ATLs) play a key role in conceptual knowledge representation. The hub-and-spoke theory suggests that the contribution of the ATLs to semantic representation is (a) transmodal, i.e. integrating information from multiple sensorimotor and verbal modalities, and (b) pan-categorical, representing concepts from all categories. Another literature, however, suggests that this region's responses are modality- and category-selective; prominent examples include category selectivity for socially relevant concepts and face recognition. The predictions of each approach have never been directly compared. We used data from three studies to compare category-selective responses within the ATLs. Study 1 compared ATL responses to famous people versus another conceptual category (landmarks) from visual versus auditory inputs. Study 2 compared ATL responses to famous people from pictorial and written word inputs. Study 3 compared ATL responses to a different kind of socially relevant stimuli, namely abstract non-person-related words, in order to ascertain whether ATL subregions are engaged for social concepts more generally or only for person-related knowledge. Across all three studies a dominant bilateral ventral ATL cluster responded to *all* categories in *all* modalities. Anterior to this ‘pan-category’ transmodal region, a second cluster responded more weakly overall yet selectively for people, but did so equally for spoken names and faces (Study 1). A third region in the anterior superior temporal gyrus responded selectively to abstract socially relevant words (Study 3), but did not respond to concrete socially relevant words (i.e. written names; Study 2). These findings can be accommodated by the graded hub-and-spoke model of concept representation. On this view, the ventral ATL is the centre point of a bilateral ATL hub, which contributes to conceptual representation through transmodal distillation of information arising from multiple modality-specific association cortices. Partial specialization occurs across the graded ATL hub as a consequence of gradedly differential connectivity across the region.

This article is part of the theme issue ‘Varieties of abstract concepts: development, use and representation in the brain’.

## 1 . Introduction

The neural organization of conceptual knowledge (or semantic knowledge) has long been a fundamental issue in cognitive neuroscience, with much debate on the degree to which representations are segregated by modality and category. On the one hand, researchers have emphasized cortical specialization for specific modalities and categories of knowledge [1–5]. Other researchers, while not denying these specializations, have argued that true conceptual knowledge additionally requires a transmodal level of representation that integrates across modalities and possibly categories [6–9]. Recent neuroimaging studies using multivariate techniques have also identified brain regions that process transmodal semantic information [10–12]. Here, we investigated the organization of knowledge in the anterior temporal lobes (ATLs), a region that has emerged as a key contributor to conceptual representation [10,13–17].

Currently, there are different literatures that propose contrastive hypotheses about the ATLs, yet their predictions have never been directly compared. The hub-and-spoke theory holds that the ATLs form a pan-category transmodal ‘hub’ that develops coherent conceptual representations through interaction with distributed information sources [7,8,14,17,18]. This theory stems from studies of semantic dementia (SD) patients who exhibit a selective yet progressive multimodal, pan-category impairment of semantic knowledge, following bilateral ATL atrophy [19–21]. Performance on semantic tasks in SD patients is correlated with the amount of atrophy and hypometabolism in the ventrolateral ATLs [22]. SD patients exhibit generalized deficits across different conceptual categories, including concrete and abstract words [23–25], living and non-living items [26,27], and people [28]. Recent fMRI, rTMS and subdural grid-electrode explorations also directly implicate the ATLs as a transmodal, pan-category hub [13,15,29–32].

Conversely, a separate literature proposes that the ATLs are involved in processing socially relevant semantic cognition [33–36]. This account is consistent with long-standing observations that the ATLs are part of a wider network involved in social cognition in humans and primates [37–41]. The question of what constitutes a ‘social concept’ is an important one, and remains relatively ill-defined in the literature. Within the existing literature ‘social cognition’ encompasses topics such as (but not limited to) recognizing conspecifics (people, most commonly from a face) [42–48], processing socially relevant words [33,41,49], recognizing emotions [50–53] and understanding the intention of others (theory of mind; [40,54,55]). In this paper, we used the term ‘socially relevant concept’ to refer to semantic information which has social connotations/implications. While the definition of socially relevant concepts remains broad and ill-defined, several groups have proposed that all or part of the ATLs selectively code social concepts, including person (face) knowledge and emotional concepts [34,36,41,49,56,57]. Indeed deficits in social behaviour are often observed in SD patients, including social awkwardness, person recognition deficits and a loss of empathy [58–61]. These findings could reflect either a dedicated role of ATL regions in social concepts and/or the contribution that a more generalized ATL semantic system might play in activation of all concepts including social items. In a novel extension from the clinical findings to fMRI, Zahn *et al.* [41] demonstrated that activation associated with socially related words (e.g. polite) versus non-social words (e.g. nutritious) was localized to the right anterior superior temporal gyrus (aSTG) in neurologically intact participants. However, a direct replication of the Zahn *et al.* [41] task found greater activation for social > non-social words in the left aSTG, rather than in the right aSTG [33], suggesting that both ATLs may play a role in the task. This finding of differential activation in the aSTG for social concepts was replicated in a recent study which employed more stringent matching of the stimuli [62]. Indeed the potential role of the left as well as the right ATL in social concepts was underlined by the study of Chan *et al.* [59], which, in a formal exploration, found social and behavioural deficits in both left > right and right > left SD patients (with a greater proportion of right > left, albeit more severe, SD patients showing social and behavioural deficits).

Potentially related to the argument that the ATLs show a category effect for socially relevant concepts, a third literature proposes that the ATLs are selectively involved in face processing [56,63–67], perhaps in the function of linking familiar faces to stored semantic knowledge [68]. In support of this, congenital prosopagnosia has been linked to reduced (ventral) ATL volume, and damage to the right ATL can result in greater deficits in face recognition than for other categories [69–72]. Likewise, some fMRI studies have shown that the ATLs bilaterally (though more commonly in the right hemisphere) respond more to faces than non-face objects [46,63,65]. This face-related ATL activation has been proposed to be the human homologue to the ‘anterior temporal face patches' recently observed in macaques [47,64,73–75]. However, the necessarily selective focus on face processing in these studies means that their results are based solely on visual stimuli. Therefore, it is unclear whether the face-related ATL region responds selectively to faces or to transmodal person knowledge [45].

These neuroimaging datasets—general semantics versus social semantics versus face representation—have emerged in parallel and thus a critical question that arises is whether they report activations in the same or different regions within the ATL. Formal analysis of the current literature does allow us to answer this question. Specifically, in table 1, we report peaks from a number of studies investigating either general semantic knowledge or face representation. The two sets of studies report rather similar (and typically bilateral) peaks, although peaks from the face-related studies are, on average, more anterior/medial along the ventral surface (approx. 1 cm away). Based on these data, it is difficult to distinguish between two interpretations: (1) that faces activate the same ATL regions as other meaningful stimuli but perhaps do so more strongly, or (2) that there are subdivisions within the ATLs which respond differently, with a more anterior/medial area being face-selective.

**Table 1.** Peak MNI coordinates taken from the general semantics literature and face-selective literature.

	**LH**			**RH**			
study	***X***	***Y***	***Z***	***X***	***Y***	***Z***	**contrast**
general semantics							
Devlin *et al.* [76]	−42	−14	−28				semantic > letter categorization
Sharp *et al.* [77]	−38	−18	−32				speech > vocoded speech
Binney *et al.* [13]	−36	−15	−30				semantics (words) > numbers
	−39	−9	−36				semantics (words) > numbers
	−39	−24	−24				semantics (words) > numbers
Visser *et al.* [78]	−36	−14	−40	40	−8	−38	semantic (words) > letters
				34	−12	−40	semantic (words) > letters
				58	−20	−26	semantic (words) > letters
				52	−8	−40	semantic (words) > letters
Visser *et al.* [31]	−36	−9	−36	35	−5	−36	semantic (pictures, auditory words, environmental sounds) > control
Visser *et al.* [30]	−57	−15	−24				semantic (pictures + words) > control
Hoffman *et al.* [29]	−42	−14	−34				synonyms > numbers
Jackson *et al.* [79]	−45	−15	−27				semantic task > letter matching
face-selective ATL							
Kriegeskorte *et al.* [65]				42	0	−48	face 1 > face 2
Nestor *et al.* [80]				50	−9	−28	face individuation (face 1 versus face 2)
Pinsk *et al.* [75]	−38	−17	−30	42	−1	−39	faces > objects
Nestor *et al.* [46]				19	6	−26	face individuation
Nasr *et al.* [81]	−33	−7	−33	32	−2	−36	normal faces
				34	−8	−36	faces > places
Axelrod *et al.* [63]	−34	−11	−35	34	−10	−39	faces > objects (tables)
Avidan *et al.* [69]	−34	−4	−34	34	−2	−42	faces > buildings
Goesaert *et al.* [82]	−33	−8	−33	33	−8	−33	faces > objects
Mur *et al.* [83]	−26	−6	−27	35	−3	−25	faces (learned unfam faces) > baseline [rest]
Von der Heide *et al.* [84]	−50	−10	10	54	−4	−8	famous faces > baseline (ALE)
	−46	6	−22				familiar faces > baseline (ALE)
	−52	−8	−10	52	−2	−8	famous > familiar faces (ALE)
	−44	4	−24				
	−28	−8	−22				
	−41	9	−29	32	6	−26	faces > landmarks (empirical study)
	−37	4	−31	45	4	−26	
	−32	17	−29				famous faces > novel faces
	−30	10	−24	25	6	−24	famous faces > novel landmarks
	−38	20	−25				famous faces > familiar faces
Fairhall & Caramazza [85]	−60	−10	−29				knowledge about person kinds
	−57	−10	−14				localizer: famous people (faces) >control >> famous places > control
Fairhall, Anzellotti, Ubaldi, & Caramazza [4]				60	−4	−26	people > place
Anzellotti & Caramazza [86]	−37	6	−25	41	6	−22	face individuation (MVPA)
Elbich *et al.* [87]	−37	−4	−25	33	−1	−25	extended face region—taken as voxels closest to those reported in a previous study [83]
Yang *et al.* [88]	−41	1	−41	44	1	−37	faces > objects
Pinsk *et al.* [75]	−62	−7	−19	57	−10	−16	faces > objects
Harry *et al.* [67]	−39	−13	−33	37	−14	−39	mean MNI peaks from ROI analysis

This study was designed specifically to draw these three currently separate literatures together in order to understand the role of various ATL subregions in the representation of different kinds of social versus non-social concept. Specifically, we conducted the first comparison of ATL responses to different kinds of socially relevant concepts using three datasets which all used neuroimaging sequences tailored to acquiring signal in the ATL and used appropriate control conditions. First, we compared ATL activation to people versus another conceptual category across different modalities (Study 1). Next, we verified the ATL responses to people-related knowledge using another modality of presentation (famous names presented as written words; Study 2), in order to replicate and extend the findings of Study 1 in a separate dataset. Finally, we compared activation within the ATLs to different classes of socially relevant concepts (e.g. socially relevant words) to assess whether activation to socially relevant concepts is consistent or whether there is selective activation for social knowledge versus other kinds of semantics (Study 3). We also compared activation within the ATL between abstract socially relevant words versus concrete socially relevant words (i.e. famous names from Study 2).

## 2. Method

**Figure 1.**
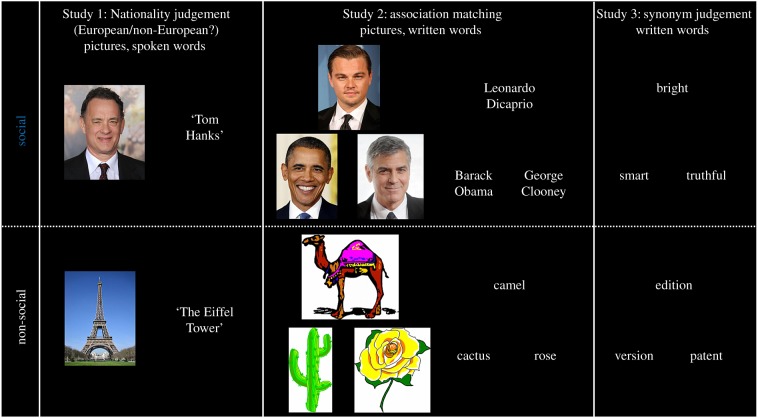
Social and non-social semantic conditions included in Studies 1–3.

We compared data from three studies, each exploring different examples of socially relevant concepts (figure 1). Two of the three studies explored the perception/representation of person knowledge (Study 1 and Study 2), and one study explored written words depicting (abstract) socially relevant concepts (Study 3). Study 1 was used to compare socially relevant concepts (faces, spoken names) versus a well-established control condition (landmarks) to identify socially relevant activations in the ATLs. Study 2 was used to verify whether the findings of Study 1 could be replicated and extended to another modality of presentation (famous names presented as written words). Study 3 was used to assess whether the findings from famous people generalized to other socially relevant stimuli (i.e. socially relevant concept words).

### (a) Stimuli and tasks

Data were collected from three separate fMRI studies (*n* = 59). All of the participants in the three studies were unique. Each study consisted of at least one social semantic condition, one non-social semantic condition from the same modality, and a modality-matched non-semantic control task. All three studies used a PC running the E-Prime software (Psychology Software Tools, Pittsburgh, PA) for presentation of stimuli and recording of responses. For behavioural results across all three studies, see electronic supplementary material, table S1.

#### (i) Study 1: stimuli, tasks and procedure

Study 1 (*n* = 20) consisted of pictures and spoken names of famous people and famous landmarks. Landmarks were chosen as comparison categories for people because landmarks are highly prominent within the visual perception literature as a contrast for faces and, like faces, are also classified as ‘unique entities’ [89]. Study 1 also contained data from a third non-unique conceptual category (animals); however, these data will not be discussed here. Each conceptual category (people, landmarks) contained 72 stimuli, which were presented twice during scanning, once as a picture and once as a spoken name. Stimuli were presented in two modalities to address a discrepancy in the literature: studies proposing that the ATLs are involved in face processing have exclusively used visual stimuli and do not make explicit predictions about whether this area is visually selective or transmodal [63,65]. This stands in contrast to the general semantic literature which provides evidence that the ATLs respond across multiple modalities for multiple categories [30,31,90]. Visual and auditory control conditions were used to account for low-level sensory effects and to provide an attention-demanding baseline condition, which is a crucial factor for observing ATL activations [91,92]. The visual control items were generated by scrambling 72 images from the three conceptual categories; these were created using the Java Runtime Environment (www.SunMicrosystems.com) by scrambling each image into 120 pieces and rearranging them in a random order. The auditory control condition consisted of 6 phase-scrambled auditory tones. Stimuli were presented in blocks of the same condition to participants in the scanner and the task was a nationality judgement task (Is the stimulus European or Non-European?). For the control conditions, participants were used to make ‘high/low’ decisions for each stimulus (Is the scrambled image high or low on the screen?’, ‘Is the tone high or low in pitch?). To ensure the semantic and control tasks were matched for eye movements, the visual semantic conditions were also randomly presented above or below the fixation cross.

Participants completed three functional scans, with a total scan time of 36 min. During scanning, stimuli were presented in a block design. Each functional scan contained alternating blocks of visual or auditory stimuli from one condition; half of the runs started with an auditory block (A – V – A – V) and half the runs started with a visual block (V – A – V – A); this order was counterbalanced across participants. Each block contained 6 trials. Each stimulus was presented sequentially and in isolation for 2500 ms, with an inter-stimulus interval (ISI) of 500 ms. The eight experimental conditions (6 semantic + 2 control conditions) were sampled 12 times in a counterbalanced order, giving a total of 96 blocks. At the start of each block, a written word probe prompted participants as to which task was coming up. Visual stimuli were presented via a mirror mounted on the head coil, angled at a screen at the foot of the scanner bed. Auditory stimuli were presented via noise cancelling headphones (MkII+ headphones, MR confon GmbH; http://www.mr-confon.de/en/) in conjunction with ear plugs, to reduce scanner noise. To ensure that the auditory stimuli were intelligible for each participant, practice trials were run while the scanner was active and the sound level was adjusted as necessary.

#### (ii) Study 2: stimuli, tasks and procedure

Study 2 (*n* = 20) also involved semantic judgements regarding famous people, this time incorporating pictures and written words (names). On each trial participants were presented with a probe item and asked to decide which of the two alternatives shared the same occupation. Each stimulus triad was presented simultaneously. In each triad all stimuli came from the same gender and nationality. Alongside this condition there was a non-social semantic association task, consisting of a variant of the widely used Camel and Cactus test [20]; here the task was to pick which of the choice items is associated with the probe item. To match the occupation matching task, items were presented either as pictures (CCp) or written words (CCw). Different items were used in the word and picture versions of the Camel and Cactus and occupation matching task to avoid priming effects. Each condition consisted of 33 trials. Again, modality-specific non-semantic control tasks were included in Study 2, namely scrambled versions of the famous faces/names and Camel and Cactus pictures/words. Participants were instructed to choose which of the choice items was identical to the probe. The data reported here form part of a larger study comparing brain activation in control participants to a set of post-surgical temporal lobe epilepsy (TLE) patients. As part of the larger study, control participants saw each of the semantic conditions twice, once at a speed typical for a healthy population (‘standard speed’; 2.5 s/triad) and once at a slower speed (5 s/triad); although importantly the items used in both scans were different to avoid priming effects (i.e. items which were presented as a picture in the ‘standard’ speed scan were shown as written words in the ‘slower’ speed scan). The slower speed was used in relation to the behavioural slowing seen in the patient group and thus to allow direct comparison between the data from the patients to the control group. For the purposes of the current reanalysis only the ‘standard’ blocks were entered into the analysis to match the task demands to the other studies.

Study 2 consisted of four functional scans, each with a total scan time of 8.45 min. During scanning, stimuli were presented in a block design. Each functional scan contained stimuli from one semantic condition (CCw, CCp, famous names or famous faces) and from the relevant baseline condition (scrambled pictures or scrambled words). This was done to avoid task-switching effects in the scanner. Each block contained three trials from one experimental condition. Each stimulus and the response screen were presented for 5000 ms, with an ISI of 500 ms. The two experimental conditions (semantic and baseline) were sampled 11 times per functional scan in a counterbalanced order, giving a total of 22 blocks per scan. The order of the scans was randomized and counterbalanced across participants. Stimuli were presented visually via a mirror mounted on the head coil, angled at a screen at the foot of the scanner bed. All participants underwent practice trials before beginning the scan to familiarize them with the tasks.

#### (iii) Study 3: stimuli, tasks and procedure

The data from Study 3 (*n* = 19) were taken from a previously published investigation of socially relevant concepts in the ATL [62]. Briefly, Study 3 presented participants with written synonym judgement decisions. Stimuli were either socially relevant concept words (e.g. bright) or non-social abstract concept words (e.g. edition), matched closely for psycholinguistic properties including frequency, imageability and semantic diversity (see Binney *et al.* [61,62] for full details of stimulus matching). Each condition consisted of 48 triads. In all conditions participants were instructed to choose which of the two choice words was associated with the probe word. The non-semantic control condition was a number judgement task; a triad of numbers was presented on screen and participants were instructed to choose which of the two choice numbers was closer in numerical value to the probe number.

For Study 3 a block design was used, each block lasting 13.5 s and consisting of three trials from the same experimental condition. Each trial began with a fixation cross presented in the centre of the screen for 500 ms, followed by a stimulus triad (probe and choice words simultaneously). The stimuli remained on the screen for a fixed duration of 4000 ms after which the next trial began. Participants responded by pressing one of two buttons on an MR-compatible response box. Study 3 consisted of two 15 min functional runs separated by a 10 min interval. Each run contained 16 blocks of the number judgement task and 16 blocks of the three semantic judgement conditions. All conditions were presented in a pseudo-random order.

### (b) Scanning

#### (i) Imaging parameters

Traditionally, imaging the ventral ATLs has been problematic because of a number of technical issues including the nature of the baseline contrast tasks as well as gradient-echo EPI signal dropout and distortion [76,92]. These issues have been tackled through recent methodological developments [78,93]. Across all three studies reported here, the core semantic task was contrasted against an active baseline (see above) using either dual-echo EPI imaging Study 1 + 2 [94] or spin-echo EPI imaging Study 3 [93] to improve signal in the ATLs.

For Study 1 and 2 all scans were acquired on a 3T Phillips Achieva scanner, with a 32-channel head coil with a SENSE factor of 2.5. A dual-echo EPI sequence was used to improve the signal-to-noise ratio (SNR) in the ATLs [94]. Using this technique, each scan consisted of two images acquired simultaneously with different echo times: a short echo optimized to obtain signal from the ATLs and a long echo optimized for good whole-brain coverage. The sequence included 31 slices covering the whole brain with repetition time (TR) = 2.8 s, echo times (TE) = 12 and 35 ms, flip angle = 85^o^, FOV = 240 × 240 mm, resolution matrix = 80 × 80, slice thickness = 4 mm and voxel size = 3 × 3×4 mm. All functional scans were acquired using a tilt, up to 45° off the AC–PC line, to reduce ghosting artefacts in the temporal lobes. In Study 1, functional scans were collected in three 12 min runs; each run acquired 255 dynamic scans (including two dummy scans, which were excluded). In Study 2, functional scans were collected in four 4.3 min runs; each run contained stimuli from one of the four semantic conditions (faces, written names, CCp, CCw) and one of the modality-appropriate non-semantic control conditions and acquired 88 dynamic scans (including two dummy scans, which were excluded). To address field inhomogenities, a B0 field-map was acquired using identical parameters to the functional scans except for the following: TR = 599 ms, TEs = 5.19 and 6.65 ms. A high-resolution T1 weighted structural scan was acquired for spatial normalization, including 260 slices covering the whole brain with TR = 8.4 ms, TE = 3.9 ms, flip angle = 8°, FOV = 240 × 191 mm, resolution matrix = 256 × 206, voxel size = 0.9 × 1.7 × 0.9 mm.

Study 3 used spin-echo data acquisition combined with post-acquisition distortion correction [93]. This imaging sequence has been used previously to demonstrate robust ATL activation for a variety of semantic tasks [13,29,31,78,95]. All scans for Study 3 were acquired on a 3T Philips Achieva scanner using an 8 element SENSE head coil with a sense factor of 2.5. The spin-echo EPI fMRI sequence included 31 slices covering the whole brain with echo time (TE) = 70 ms, time to repetition (TR) = 3200 ms, flip angle = 90°, 96 × 96 matrix, reconstructed resolution 2.5 × 2.5 mm and slice thickness 4.0 mm. 550 images were acquired in total, collected in two runs of 15 min each. Following the method of Embleton *et al*. [93] for distortion-corrected spin-echo fMRI, the images were acquired with a single direction k space traversal in the left–right phase encoding direction. In between the two functional runs, a brief ‘pre-scan’ was acquired, consisting of 10 volumes of dual direction k space traversal SE EPI scans. This gave 10 pairs of images matching the functional time series but with opposing direction distortions (10 left–right and 10 right–left). These scans were used in the distortion correction procedure (see below). A high-resolution T2-weighted turbo spin-echo scan with an in-plane resolution of 0.94 mm and slice thickness of 2.1 mm was obtained as a structural reference to provide a qualitative indication of distortion correction accuracy. In addition, a high-resolution T1-weighted 3D turbo field echo inversion recovery image was acquired (TR ≈ 2000 ms, TE = 3.9 ms, Inversion time (TI) = 1150 ms, flip angle 8°, 256 × 205 matrix reconstructed to 256 × 256, reconstructed resolution 0.938 × 0.938 mm and slice thickness of 0.9 mm, SENSE factor = 2.5), with 170 slices covering the whole brain. This image was used for estimating transforms to warp functional images into standard stereotactic space. Full details of the distortion correction technique and preprocessing steps for Study 3 can be found here [62].

#### (ii) fMRI data analysis

For all three studies, data were analysed to compare the ‘social’ conditions to the ‘non-social’ conditions in the dataset (figure 1). For Study 1, the social condition was the faces and spoken names of famous people, and the non-social condition was pictures and spoken names of famous landmarks. For Study 2 the social condition was the faces and written names of famous people, and the non-social condition was the picture and word version of the Camel and Cactus test. For Study 3 the social condition was the socially relevant concept words, and the ‘non-social’ condition was the abstract non-social concept words.

**Figure 2.**
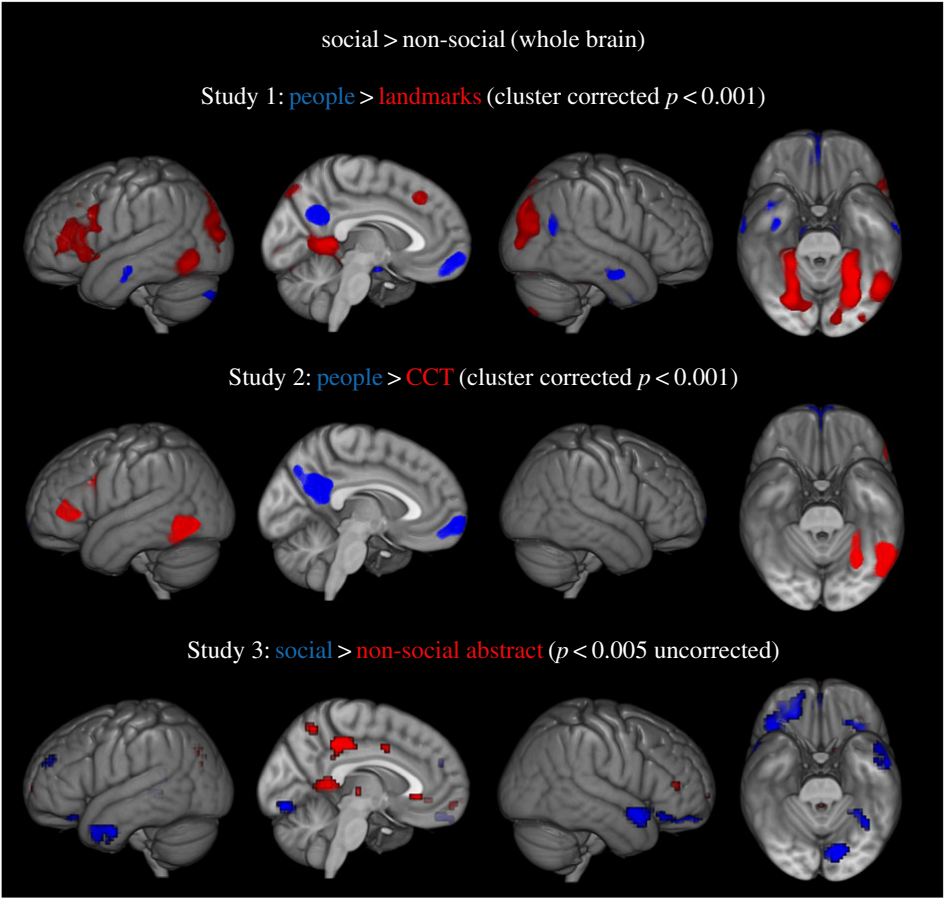
Whole-brain analysis of Studies 1–3. Regions in blue show stronger activation for social > non-social semantic conditions, regions in red show stronger activation for non-social versus social semantic conditions.

Data were motion-corrected and co-registered to the anatomical T1. Images were also spatially normalized to the MNI standard space and resampled to 3 × 3 × 3 mm dimensions, and smoothed using an 8 mm Gaussian FWHM kernel. First- and second-level analyses were carried out using SPM8 (Wellcome Department of Imaging Neuroscience, London; www.fil.ion.ucl.ac.uk/spm). At the first level, data for each study were entered into separate general linear model analyses by modelling each condition (social, non-social, non-semantic control) as a separate regressor using a boxcar function convolved with the canonical haemodynamic response function. Contrasts were calculated for each condition (social, non-social) versus the modality-relevant non-semantic control condition. At the second level, the data from each study were entered into separate one-way ANOVA models. The contrasts of interest were social > non-social semantics in each of the three studies (figure 2). ‘Social > non-semantic baseline’ + ’Non-Social > non-semantic baseline’ contrasts were also calculated at the second level (electronic supplementary material, figure 1). Unless otherwise stated, for Studies 1 and 2 a voxel height threshold of *p* < 0.001, cluster-corrected using an FWE *p* < 0.05 was used. For Study 3 an uncorrected voxel height threshold of *p* < 0.005 was used as per the originally reported results [62].

To explore differential activation across a set of ATL regions for different categories of social information, we created four *a priori* ROIs using the Marsbar toolbox [96]; each ROI was 6 mm in diameter. The first ROI was a region commonly activated in functional imaging studies of semantic cognition [13,31,78]. This ventral ATL ROI [MNI: −36 −15 −30; 36 −15 −30] was localized in Binney *et al.* [13]. The next two regions were chosen because they are often reported in studies investigating the role of the ATL in face processing: (a) the temporal pole (TP), a region slightly anterior and medial to the vATL ROI and (b) the anterior middle temporal gyrus (aMTG), a region on the lateral surface of the ATL. Both the TP (ROI no.2) and aMTG (ROI no.3) were localized from a meta-analysis of 17 studies investigating face recognition in the ATLs (coordinates reported in table 1). We used activation likelihood estimation (ALE) analysis [97], a method that extracts coordinates from a set of neuroimaging studies and estimates the likelihood of activation across each voxel in the brain. The resultant ‘activation likelihood maps' can then be viewed on a standard brain. The ATL peaks from 17 ‘face-selective’ studies ( table 1) were entered and an overall activation likelihood map was generated to show ATL coverage. This was thresholded using a false discovery rate (FDR) of *p* < 0.05 to correct for multiple comparisons. Four peak MNI regions of activation likelihood were extracted (TP ROI no. 2: −37 4 −29; 31 1 −25, aMTG ROI no. 3: −59 −7 −18; 61 −1 −16). The fourth *a priori* ROI was the aSTG [MNI: −51 16 −27; −51 16 −27]; this subregion of the ATL has been previously associated with social processing [41,57,62]. Coordinates were taken from Ross & Olson [33] using a contrast comparing social versus animal concept words. The coordinates were converted from Talariach to MNI space using the tal2icbm_spm.m function.

## 3. Results

### (a) Whole-brain analysis: are there regions of the anterior temporal lobe which respond more to socially relevant concepts?

First, we investigated whether there were subregions of the ATLs which responded more to socially relevant concepts compared to other types of semantic information. Regions involved in socially relevant semantic knowledge were identified using the whole-brain contrast social > non-social semantics in each of the three datasets separately. Peak activations for each study are listed in table 2. Figure 2 shows a network of regions activated by the socially relevant semantic conditions (blue) across the three datasets. For Study 1 person-related clusters were primarily localized in the right hemisphere, including the ventral aspect of the ATL/TP, precuneus, orbitofrontal cortex, hippocampus, anterior middle temporal gyrus (aMTG) and the temporo-parietal junction (table 2). These regions (with the exception of the orbitofrontal cortex and ATL) are in line with the findings from previous studies of conceptual category representation, which showed transmodal responses to person knowledge in the precuneus [4,85], suggesting these regions may play a specific role in processing more socially salient semantic knowledge. No other category differences were localized in the ATLs (i.e. non-social > social). Activation to transmodal landmarks were widespread, and included bilateral parahippocampal gyri, precuneus, lateral occipital cortex and left inferior frontal gyrus (table 2).

**Table 2.** Peak coordinates from the whole-brain analysis across each of the three datasets.

		MNI				
contrast	region	*X*	*Y*	*Z*	extent	*Z*-value
Study 1—cluster corrected, *p* < 0.001 uncorrected						
social (face + spoken name) > non-social (picture + spoken landmark name)		5	−56	25	7123	5.32
		58	−61	18	2740	5.32
		16	−11	−16	3745	5.05
		26	−9	−17		4.93
		13	−19	−11		3.22
		37	8	−36	3072	4.80
		38	−4	−39		3.77
		39	−12	−41		3.63
		2	60	−12	5641	4.65
		−23	−13	−16	2647	4.63
		−29	1	−15		3.75
		−20	−89	−41	1622	3.99
		−33	−82	−36		3.98
		52	−17	−16	2115	3.97
		64	−4	−21		3.51
		−62	−14	−18	926	3.93
		−64	−11	−27		3.56
		8	63	11	1243	3.90
non-social (picture + spoken landmark) > social (face + spoken name)		28	−47	−12	127 117	Inf
		−26	−47	−15		7.77
		22	−41	−20		7.37
		30	−74	−53	1216	4.95
		−48	27	15	16 532	4.89
		−50	38	4		4.63
		−49	24	−3		4.42
		−3	28	43	1565	4.34
		−22	6	46	1092	3.93
Study 2—cluster corrected, *p* < 0.001 uncorrected						
social (face + written name) > non-social (CCp + CCw)	Precuneus	3	−52	20	13 793	5.14
		1	−72	38		3.91
		5	−65	44		3.73
	orbitofrontal cortex	−3	63	−7	4613	5.10
non-social (CCp + CCw) > social (face + written name)		−48	−51	−13	9741	5.34
		−50	−68	−10		4.88
		−56	−71	−15		3.82
		−28	−55	−16	3276	4.67
		−32	−36	−20		3.52
		−49	36	0	2715	4.46
		−56	30	−3		3.61
		−42	36	18		3.41
		−45	7	23	3538	4.14
		−50	12	27		4.06
		−37	4	23		3.81
Study 3—*p* < 0.005 uncorrected (min voxel size = 10)						
social concept words> non-social abstract words	anterior middle temporal gyrus	57	9	−15	77	4.34
	orbitofrontal cortex	21	45	−18	96	4.13
		42	33	−18		3.84
		36	51	−18		3.36
	anterior inferior temporal gyrus	−54	9	−33	80	4.08
		−60	−3	−33		3.25
		−51	−3	−36		3.06
	medial frontal cortex	−36	51	24	15	3.98
	lingual gyrus	−12	−78	−12	79	3.87
	posterior superior temporal gyrus	−57	−42	15	32	3.84
	medial occipital gyrus	−18	−93	6	28	3.60
	posterior middle temporal gyrus	−60	−39	0	27	3.43
	middle temporal gyrus	−45	−27	−9	14	3.40
	posterior fusiform gyrus	24	−78	−33	26	3.29
	post-central gyrus	27	−33	60	17	3.28
	calcarine sulcus	15	−87	3	53	3.26
		12	−78	0		3.12
	superior medial frontal cortex	−9	48	27	15	3.23
		−12	60	24		2.74
	inferior frontal gyrus (orbitalis)	−36	30	−21	19	3.20
		−27	33	−21		2.82
	posterior fusiform gyrus	−36	−54	−18	25	3.15
		−30	−45	−21		2.88
	gyrus rectus	3	45	−18	25	2.95
non-social abstract words > social concept words		−27	39	−9	53	4.02
		−9	57	−9	15	3.99
		−3	−33	42	84	3.80
		−42	−75	36	18	3.59
		−45	−78	24		2.99
		−3	−45	6	77	3.44
		−6	−54	6		2.94
		−27	−42	3	50	3.25
		−33	−60	3		2.91
		−15	3	0	33	3.22
		−9	−63	54	24	3.10
		−12	−51	51		2.72
		48	39	9	10	3.07
		21	−42	6	11	3.06
		−27	60	6	11	2.93

For Study 2, an identical pattern of activation was observed in the midline structure of the orbitofrontal cortex and the precuneus; however, at this threshold there were no significant clusters in the temporo-parietal junction or the ATL. Activation to the CCT was localized to the left posterior temporal cortex, left lateral occipital cortex and left inferior frontal gyrus. Study 3 also showed stronger activation for socially relevant concepts in the left temporo-parietal junction, including the supramarginal gyrus and the posterior MTG, which extended into the posterior insula cortex. There were also two medial occipital clusters, a left hemisphere cluster in the superior aspect of the cuneus and a bilateral cluster peaking at the lingual gyrus. There was also a cluster in the left inferior frontal gyrus.

Across all three studies there was significant overlap across the ATL and the brain more widely (pink) between the contrasts ‘social > control’ and ‘non-social > control’ (electronic supplementary material, figure S1) when comparing the conditions of interest over the non-semantic baseline, providing support for the hypothesis that both types of semantic information are processed by similar subregions of the ATL.

### (b) ROI analysis: do anterior temporal lobe subregions respond to transmodal person knowledge or face information?

Next, we investigated whether the ATL subregions identified in the whole-brain analysis responded selectively to *transmodal* person knowledge, based on previous research showing that the ATL is activated by famous names as well as faces [44,45]. To do this, we used *a priori* ROI analysis, using peaks taken from the previous literature. Data from Study 1 and Study 2 were analysed using 2 category (social, non-social) × 2 modality (picture, spoken/written) ANOVAs in each region of interest.

**Figure 3.**
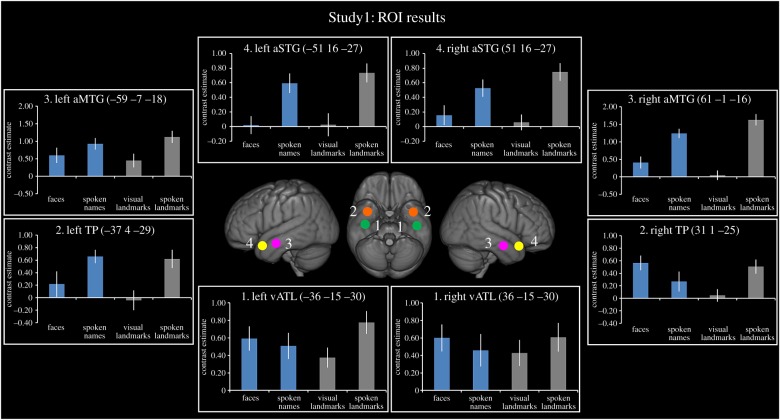
ROI analysis results for Study 1. Results are shown for four ROIs derived from the literature. Blue bars represent the social conditions and grey bars represent the non-social conditions. All bars show the relative activation for each condition of interest compared to its matched non-semantic control condition. Error bars show standard error.

Figure 3 shows a gradient of activation across the ATLs in Study 1. This functional gradient progresses from a transmodal, pan-category response in the vATL (figure 3, ROI 1) to a modality-selective (auditory) response in the aSTG (figure 3, ROI 4). The *a priori* vATL ROI (no.1) responded to both social and non-social category information in equal measures, as well as visual and auditory information. In line with this, the category×modality ANOVA showed no significant main effects of category or modality in either hemisphere. This replicates previous findings of transmodal responses in the vATL [30,31]. However, there was a significant category×modality interaction in the left vATL (*F*_1,19_ = 14.90, *p* = 0.001). This interaction may be driven by an intrinsic word length effect for the names of landmarks versus people (16.2 characters versus 12.7 characters; *t*_71_ = 5.31, *p* < 0.001); this intrinsic nature of the stimuli could have increased the difficulty of processing for the names of landmarks leading to a greater activation. No significant interaction was found in the right vATL.

In contrast to the transmodal, pan-category results in the vATL, the more anterior TP ROI (no. 2), particularly in the right hemisphere, showed selective activation for faces and spoken names of people, as well as the spoken names of landmarks. In the right hemisphere, the category × modality ANOVA showed a significant main effect of category (*F*_1,19_ = 5.13, *p* = 0.04), reflecting overall increased responses to person knowledge compared to landmarks. There was also a significant category×modality interaction in the right hemisphere (*F*_1,19_ = 7.42, *p* = 0.01). In the left hemisphere, there was a main effect of modality (*F*_1,19_ = 14.64, *p* = 0.001), reflecting overall increased responses to auditory stimuli compared to visual stimuli. The peak coordinate reported here (TP peak in Study 1: −45 7 −36; 38 3 −37) aligns well with previously reported coordinates in the face-processing literature (table 1; ROI no. 2), indicating that the anterior vATL region responds to transmodal person knowledge, rather than face knowledge specifically [45].

Extending dorsally into the aMTG (ROI no.3), the same pattern of activation for faces and spoken names of people and the spoken names of landmarks remained. This was illustrated in a significant category×modality interaction in the right hemisphere (*F*_1,19_ = 11.69, *p* = 0.0003). This effect trended towards significance in the left hemisphere (*F*_1,19_ = 3.54, *p* = 0.08). In both hemispheres, there was a significant main effect of modality (left: *F*_1,19_ = 16.87, *p* = 0.0001; right: *F*_1,19_ = 48.26, *p* < 0.0001), driven by the stronger response to auditory stimuli compared to visual stimuli. Again, coordinates from this region align with those previously reported in the face-processing literature (table 1; ROI no. 3); however, the overall response to auditory stimuli may reflect this region's proximity to auditory processing areas in the superior temporal gyrus.

By contrast, in the aSTG (ROI no.4) there was no longer a category effect for social > non-social stimuli; instead this region responded selectively to the auditory conditions regardless of category (main effect of modality; left: *F*_1,19_ = 21.53, *p* < 0.0001; right: *F*_1,19_ = 10.10, *p* = 0.005). The main effect of category was not significant in either hemisphere (left: *F*_1,19_ = 1.14, *p* = 0.30; right: *F*_1,19_ = 0.71, *p* = 0.41).

The main finding from Study 1, therefore, was of two clusters in the vATLs, both transmodal in nature, one dominant area which responded to all conceptual categories, including people (figure 3, ROI no. 1), and another more anterior ‘person-related’ cluster (figure 3, TP ROI no. 2). Across the ATLs a gradation from a transmodal effect to an auditory selective response was shown, peaking in the aSTG (ROI no. 4).

### (c) Does the pattern of activation shown in Study 1 replicate across different modalities of person knowledge?

**Figure 4.**
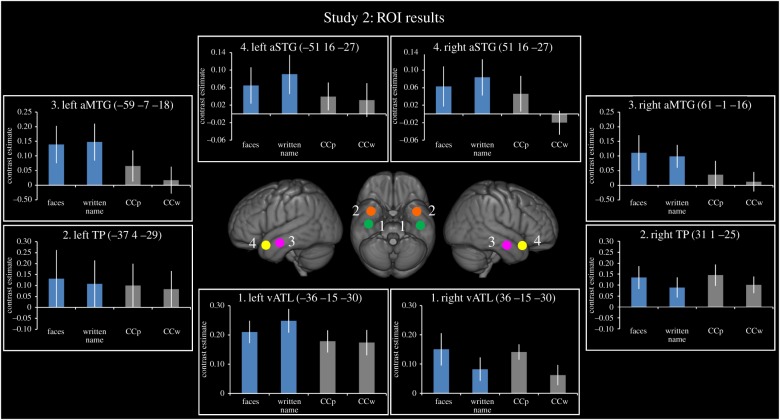
ROI analysis results for Study 2. Results are shown for four ROIs derived from the literature. Blue bars represent the social conditions and grey bars represent the non-social condition. All bars show the relative activation for each condition of interest compared to its matched non-semantic control condition. Error bars show standard error.

Figure 4 shows the ROI results for Study 2. Here, the vATL ROI showed the same pattern of activation as in Study 1—responding regardless of stimulus category and modality of presentation (picture versus written word). The only significant effect in the category×modality ANOVA was a main effect of modality in the right vATL (*F*_1,19_ = 6.34, *p* = 0.02). This was driven by reduced responses to written words (names and written versions of the Camel and Cactus) in the right hemisphere. This finding aligns with previous reports that written words produce a left lateralized response within the ATLs, whereas pictorial information produces bilateral ATL responses [98]. The only other region to show a significant effect in Study 2 was in the left aMTG, which showed a significant main effect of category (*F*_1,19_ = 8.49, *p* = 0.009); this was driven by a greater response to social > non-social stimuli. This effect trended towards significance in the right hemisphere (*F*_1,19_ = 3.69, *p* = 0.07). Critically, the aSTG, which in previous studies has shown a category effect for socially relevant (abstract) concept words [41,62], showed no significant interaction for socially relevant concrete words (i.e. famous names) in either the left (*F*_1,19_ = 0.23, *p* = 0.64) or right hemisphere (*F*_1,19_ = 1.80, *p* = 0.20).

### (d) Do anterior temporal lobe subregions also respond to different kinds of social semantic information?

**Figure 5.**
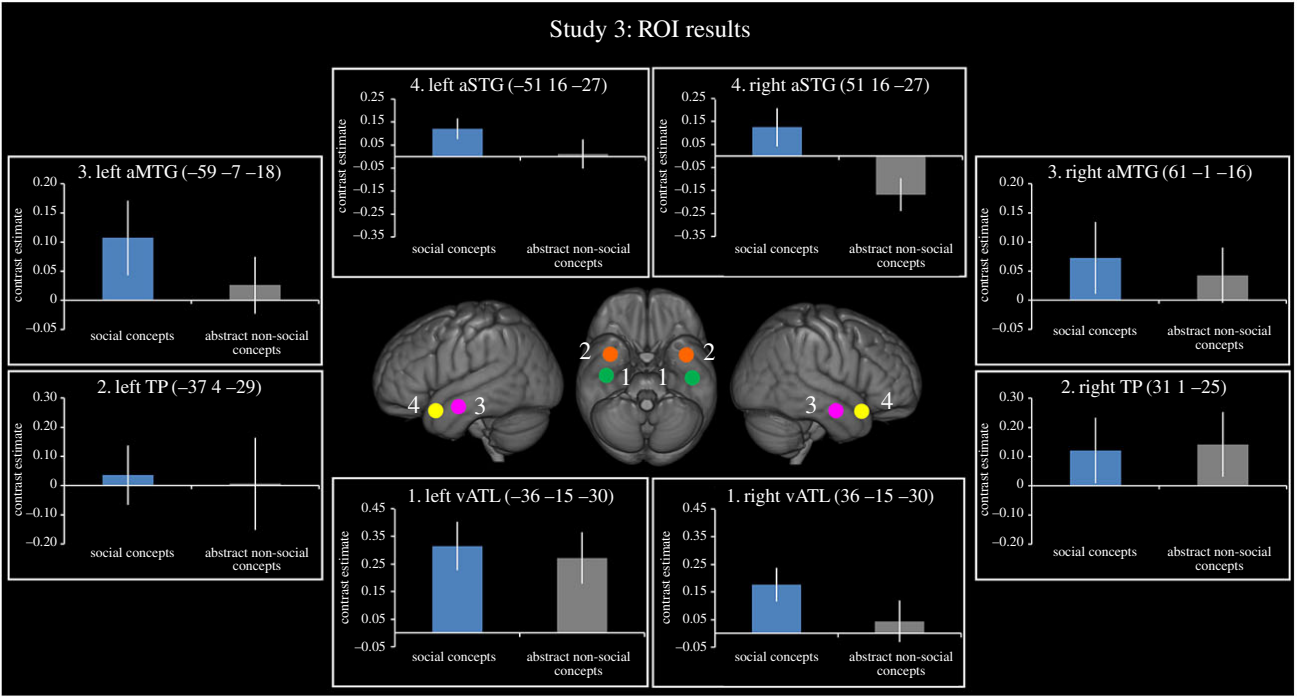
ROI analysis results for Study 3. Results are shown for four ROIs derived from the literature. Blue bars represent the social conditions and grey bars represent the non-social condition. All bars show the relative activation for each condition of interest compared to its matched non-semantic control condition. Error bars show standard error.

Finally, we asked the question whether the pattern of results shown for famous people generalize to other kinds of socially relevant semantic knowledge. For this question, data previously published comparing activation for socially relevant words [62] were plotted in the same ROIs. Paired t tests were used to compare the social versus non-social concepts. Figure 5 shows the results from Study 3. Again the vATL responded equally to social and non-social concept words (left = *t*_18_ = 0.36, *p* = 0.72; right = *t*_18_ = 1.65, *p* = 0.12), replicating the pattern of results shown in Study 1 (figure 3) and Study 2 (figure 4). The only regions which showed a category effect were the right aMTG (paired *t* test: *t*_18_ = 3.17, *p* = 0.005) and the right aSTG (paired t test: *t*_18_ = 2.72, *p* = 0.01), as reported in the original paper [62].

## 4. Discussion

This study explored the neural organization of conceptual knowledge in the ATLs. One prominent view holds that the ATLs contribute to semantic representation in a pan-category manner [7,13,14], while, in parallel, other researchers have proposed the ATLs respond selectively to socially relevant concepts [33,34,36,41] including faces [47,63,64]. For the first time, we directly compared the predictions of these different accounts of ATL function by using neuroimaging protocols that improve signal in the ATLs [92,94]. The principal finding was graded variation in ATL function. One dominant, bilateral vATL cluster responded in a pan-category and transmodal manner, overlapped with peaks reported in previous semantic studies. A second, more anterior, bilateral vATL cluster responded more weakly albeit preferentially to transmodal person knowledge and coincided with peaks reported in the face recognition literature (figure 3; tables 1 and 2). Critically, the pan-category region responded more strongly in all conditions (including person knowledge) than the anterior person-related cluster. Thus the organization of vATL function does not seem to reflect a series of mutually exclusive category-selective regions but rather one in which a dominant core vATL is joined in processing people-related knowledge by the more anterior subregion. Finally, a region in the aSTG responded to socially relevant abstract words but not to socially relevant concrete words (e.g. famous names). This region also responded to all auditory inputs in a similar manner.

These results can be accommodated by a graded version of the hub-and-spoke model of semantic representation [30,99,100]. The pan-category, transmodal responses within the core vATL accord closely with previous studies, using clinical and cognitive neuroscience methods, which implicate this area as the centre point of a transmodal representational ‘hub’ for conceptual knowledge [7,13–15,31]. On this view, the ATL-hub interacts with various distributed regions (coding modality-specific sources of information) to form coherent, generalizable concepts [7,8,13,18]. Damage to the ATLs in SD not only generates a pan-category, transmodal semantic deficit [20], but also the degree of vATL hypometabolism correlates with their level of semantic impairment [16]. Our findings also accord with multivariate neuroimaging studies showing that vATL voxels code not only the conceptual convergence of multiple sensory features e.g. colour/shape; [101] but also conceptual knowledge for different exemplars, independently of their conceptual properties e.g. how/where an object is used [102]. An important corollary of this graded hub-and-spoke theory is that the distinct vATL peaks localized here do not represent separate functional modules in the traditional sense. Instead, we believe that they are markers of continuous, graded information coding within the ATLs.

The transmodal, person-related responses in the (right) anterior vATL subregion (TP; ROI no. 2) can be accounted for by previous proposals that the ATLs are not entirely homogeneous in their function but instead develop graded specializations as a function of differential connectivity to extra-temporal regions [17,29,30,98,99,103]. According to this ‘graded’ hub-and-spoke theory, the core vATL is transmodal and pan-category because it is the centre point of multimodal inputs/outputs. Moving away from this core region, functions become increasingly influenced by one or more dominant inputs/outputs reflecting stronger connectivity to a specific neighbouring sensorimotor/verbal region [99,103]. Extending this line of argument, the *anterior* vATLs might play an important role (in addition to the core region) in representing socially relevant concepts (e.g. person knowledge), because of connections to limbic and orbitofrontal cortices via the uncinate fasciculus [104–106]. This is in line with studies indicating that temporo-polar regions contribute to the representation of social and emotional concepts [33,34,41,107]. The role of such ATL–limbic connectivity in person knowledge remains an intriguing area for future research. Consistent with this hypothesis, other structures implicated in social cognition, including the orbitofrontal cortex and precuneus, also showed transmodal person-related responses (figure 2). These regions are consistent with studies exploring conceptual category representation across the whole brain [4,85]. Importantly, the graded hub-and-spoke approach does not preclude the presence of other category-preferential responses within the ATLs, based on their particular patterns of connectivity [5,108–110].

The laterality of ATL responses to conceptual knowledge is currently highly debated [98,111–113]. Some studies indicate that patients with right ATL lesions are more likely to be prosopagnosic than those with left ATL damage [71,114,115]. Electrophysiological recordings from patients with intractable epilepsy have also revealed face-selective electrophysiological potentials in the right vATL [42]. Here, we found that activations for person knowledge in the ATLs were highly bilateral. This also follows data that patients with ATL atrophy/resection show a transmodal person deficit [28,115–117]. In addition, there were subtle hemispheric variations in the person-selective ATL regions. While the right vATL exhibited equivalent activation for faces and spoken names, the left was more active for the spoken names. This is consistent with studies suggesting that the left ATL is somewhat more important for retrieving knowledge from verbal input including people's names [28,44,116,118–121] as well as being critically involved in generating names of all types from semantic knowledge [98,108,122,123].

This study also helps to resolve another conundrum posed by the literature: the general semantics literature has suggested that the vATL is a transmodal region, whereas the face-processing literature has implicated this region, specifically, in recognition of faces. The use of visual stimuli may have been based on the assumption that the vATLs are a purely visual region because of their anatomical positioning at the apex of the visual ventral stream [124,125]. Indeed, studies have shown connectivity between the vATLs and face-selective regions in the posterior fusiform gyrus [69,70,126], and disruption of this anterior–posterior connectivity has been implicated in congenital prosopagnosia [69,70]. The transmodal responses observed here and in other studies using a variety of neuroscience methods [13,15,30,31] suggest that in addition to the strong visual input to the vATLs, it also receives input from other modalities, consistent with previous findings of transmodal responses to faces and names [45]. This study bridges, therefore, between the face-processing and semantic processing literatures by showing *transmodal* person-related vATL activation [30,31,90]. In keeping with the graded hub-and-spoke model, these findings suggest that the vATLs support the coding of coherent, transmodal semantic representations of people (alongside other categories of concept)—a proposal that accords with models of familiar face processing [68].

The responses to socially relevant abstract words in the aSTG is a highly replicable result, albeit with some debate regarding the laterality of response [33,41,49,62,127]. More recently, the causality of this region in processing social concepts has been confirmed using transcranial magnetic stimulation [57]. In this study, we were able to show that this region does not respond selectively to other kinds of socially relevant words, in particular the names of famous people (Study 2). This difference between abstract and concrete social concepts might reflect the gradient of concreteness previously shown across the ATLs [29]. In a functional imaging study the authors showed that abstract words activated aspects of the dorsolateral ATL and inferior frontal cortex relatively more than concrete words; by contrast, concrete words activated aspects of the ventromedial ATL relatively more [29]. The interpretation of this gradation was that it reflected the underlying properties of the words; concrete words are more associated with visual information, whereas abstract words are associated more with auditory–verbal information and might require greater executive control. In this study, one explanation for the result that famous names activate the vATL more may be that names of people are more intrinsically linked to a mental image of their corresponding face. This visual information may be lacking when associated with abstract words describing social concepts (e.g. polite).

In conclusion, an emerging literature suggests the vATLs exhibit face-selective responses [56,63]. Our results indicate that this picture is incomplete. An anterior vATL region does respond to images of people but does so equally strongly for their spoken names, indicating a transmodal role in the representation of person knowledge. Slightly posterior to this site, the ‘core’ vATL responds even more strongly and equally for all conceptual categories. This study provides clear evidence in favour of the ATL as a graded transmodal hub which supports coherent conceptual representation across all categories and modalities [14]. Variation of function in this region reflects graded changes in its connectivity to other brain areas, including ATL–limbic connections, which may be critical for socially relevant concepts including people. Given the inherent broad definition of what constitutes ‘social concepts’, future research should compare and contrast the activation within and across the ATLs with regard to other exemplars of socially relevant concepts.

## Data accessibility

This article has no additional data.
